# Communication from Committee on the Progress of Obstetrics, Ind. State Med. Society

**Published:** 1860-08

**Authors:** A. Heavenridge

**Affiliations:** Stilesville, Indiana


Prof. Brainard: Being appointed a Committee on the Progress of Obstetrics, at the last annual session of the Indiana State Medical Society, to report at its next annual communication, to be held in May, 1861; and as I am desirous of making as full a report as possible, I hope you will permit me to solicit information upon the following subjects, of the many readers of your journal:

The Number of Abortions.
" " Twins.
" " Stillborn.
" " Death of Mother during Labor.
" " Hemorrhage, before, during or after Labor.
" " Eclampsia, its Pathology and Treatment.
" " Rupture of Uterus.
" " Instrumental Labors.
" " Mal Presentations, and nature of.
" " Number born, and sex.
" " Puerperal Fever, its Pathology and Treatment.
" " Nursing Sore Mouth, its Pathology and Treatment.

I hope all physicians into whose hands the above may fall, will please favor me with a reply to the above interrogatories, in connection with all other matters of interest in relation to the Science and Practice of Obstetrics, by March 1st, 1861. Address.

A. HEAVENRIDGE,
Stilesville, Indiana.

